# A Cholera Case Imported from Bangladesh to Italy: Clinico-Epidemiological Management and Molecular Characterization in a Non-Endemic Country

**DOI:** 10.3390/tropicalmed8050266

**Published:** 2023-05-06

**Authors:** Valeria Russini, Maria Letizia Giancola, Grazia Brunetti, Carmela Calbi, Elena Anzivino, Carla Nisii, Lucia Scaramella, Anna Maria Dionisi, Francesca Faraglia, Marina Selleri, Laura Villa, Sarah Lovari, Maria Laura De Marchis, Teresa Bossù, Francesco Vairo, Adolfo Pagnanelli, Emanuele Nicastri

**Affiliations:** 1 Istituto Zooprofilattico Sperimentale del Lazio e della Toscana “M. Aleandri”—Sezione di Roma, 00178 Rome, Italy; valeria.russini@izslt.it (V.R.); lucia.scaramella@izslt.it (L.S.); sarah.lovari@izslt.it (S.L.); marialaura.demarchis@izslt.it (M.L.D.M.); teresa.bossu@izslt.it (T.B.); 2 National Institute for Infectious Diseases (INMI) “Lazzaro Spallanzani”, IRCCS, 00149 Rome, Italy; carla.nisii@inmi.it (C.N.); francesca.faraglia@inmi.it (F.F.); marina.selleri@inmi.it (M.S.); francesco.vairo@inmi.it (F.V.); emanuele.nicastri@inmi.it (E.N.); 3 Policlinico Casilino General Hospital, 00169 Rome, Italy; gbrunetti.polcas@eurosanita.it (G.B.); ccalbi.polcas@eurosanita.it (C.C.); elena.anzivino@gmail.com (E.A.);; 4 Department of Infectious Diseases, Istituto Superiore di Sanità, 00161 Rome, Italy; annamaria.dionisi@iss.it (A.M.D.); laura.villa@iss.it (L.V.)

**Keywords:** cholera, *Vibrio cholerae*, diarrhoea, management, Bangladesh, non-endemic, molecular characterization, whole genome sequencing, VFR

## Abstract

Despite the number of cholera outbreaks reported worldwide, only a few cases are recorded among returning European travellers. We describe the case of a 41-year-old male, returning to Italy after a stay in Bangladesh, his origin country, who presented with watery diarrhoea. *Vibrio cholerae* and norovirus were detected in the patient’s stools via multiplex PCR methods. Direct microscopy, Gram staining, culture and antibiotic susceptibility tests were performed. The isolates were tested using end-point PCR for the detection of potentially enteropathogenic *V. cholera*. Serotype and cholera toxins identification were carried out. Whole genome sequencing and bioinformatics analysis were performed, and antimicrobial resistance genes identified. A phylogenetic tree with the most similar genomes of databases previously described was built. Sample of the food brought back by the patient were also collected and analysed. The patient was diagnosed with *V. cholerae* O1, serotype Inaba, norovirus and SARS-CoV-2 concomitant infection. The isolated *V. cholerae* strain was found to belong to ST69, encoding for cholera toxin, *ctxB7* type and was phylogenetically related to the 2018 outbreak in Dhaka, Bangladesh. Adopting a multidisciplinary approach in a cholera non-endemic country ensured rapid and accurate diagnosis, timely clinical management, and epidemiological investigation at national and international level.

## 1. Introduction

Cholera is an infectious disease caused by the Gram-negative bacterial pathogen *Vibrio cholerae* of serogroups O1 and O139; they are facultative anaerobic, motile bacteria that appear to be comma shaped when observed microscopically. The disease is caused by the ingestion of contaminated food or water and is mainly related to insufficient access to potable water and inadequate hygienic conditions [1]. The incubation period is between twelve hours and five days after ingestion of contaminated food or water [2,3]. Strains of *V. cholerae* are classified into serogroups based on the structure of the outer membrane O antigen and into biotypes based on biochemical and microbiological characteristics. The seventh cholera pandemic, which is considered to be still ongoing, is caused by the El Tor biotype of *V. cholerae* of serogroup O1 (El Tor O1) [4], which replaced the “classical” biotype and spread globally since its appearance in Indonesia in 1961 [5]. *V. cholerae* O139 emerged in the early 1990s in southern India, causing diseases closely resembling *V. cholerae* O1 El Tor with large outbreaks in India and southern Bangladesh, and continued to spread across Asia until the year 2000 [6,7]. Although the aim of the roadmap planned by the Global Task Force on Cholera Control (GTFCC) of WHO [8] is to eliminate the disease in at least 20 countries by 2030 and to reduce deaths by 90% worldwide, cholera remains among the most common intestinal infectious disease with the highest morbidity and mortality rate, especially for children and older individuals.

The global burden of cholera is high and it is estimated that the number of cases is much higher than those officially notified. Based on researchers’ estimates, each year there are 2.86 million cases of cholera (ranging from 1.3 to 4.0 million), and 95,000 deaths (ranging from 21,000 to 143,000) in endemic countries worldwide, and Bangladesh is one of the countries with the highest number of cases and deaths [9]. In Bangladesh, the incidence of severe cholera in 2010 was estimated to range from 0.3 to 4.9 per 1000 population [10] and in 2015 about one out of six individuals were infected by *V. cholerae* O1 [11]. Sub-Saharan Africa accounts for the majority of this burden, but virtually all WHO regions are affected, with an estimated incidence rate ranging from 0.10 per 1000 population at risk (in AMR-B, EUR-B and WPR-B), to 25.60 in AMR-D (Haiti), and a case fatality rate ranging from 1.0% to 3.8% in both AFR-D and AFR-E [12]. In addition, diarrhoeal diseases, such as cholera, continue to pose a serious threat to the global human population and public health after natural disasters, such as earthquakes and floods [8]. While in high-income countries, owing to the access to safe water and good sanitation conditions, cholera is diagnosed as an imported and travel-related rare disease, in some low-income countries, the disease is endemic and epidemics can arise. In 2020, a large outbreak of cholera started in the Americas. Recurrent outbreaks have recently been reported in West and Central Africa with a fatality rate of about 2% [13]. The ECDC’s reporting activity suggests that cholera cases have continued to be reported in western Africa and south-east Asia over the past months and outbreaks have also been notified in the eastern and southern parts of Africa as well as in some parts of the Middle East [14]. Cholera, therefore is still a major challenge worldwide.

Despite the frequency of cholera outbreaks noted worldwide, only a few cases are reported each year among returning European travellers. The risk of cholera infection in this population is low, even though sporadic importation of cases in European countries remains possible after visiting cholera-affected areas. From 2018 to 2021, 55 cases were reported in Europe and European Economic Area (EU/EEA) countries; 26, 26, 0 and 3 cases were reported in 2018, 2019, 2020 and 2021, respectively, with at least 96% of cases linked to a recent travel history. Information on cases is not yet available for 2022. One out of fifty-five EU/EEA cases was reported in Italy in 2019 [15,16].

Herein, we describe the case of a young man returning to Italy from his home country of Bangladesh along with other family members (visiting friends and relatives—VFR), who was simultaneously affected by *V. cholerae*, norovirus and SARS-CoV-2 infection.

## 2. Materials and Methods

### 2.1. Microbiological Methods for Bacterial Identification

Stool samples from the patient were first processed in the microbiology laboratories of the “Policlinico Casilino” hospital and then at “Lazzaro Spallanzani” National Institute for Infectious Diseases where the patient was referred (both hospitals are located in Rome). *V. cholerae* and norovirus were detected using multiplex PCR methods in both laboratories (FilmArray^®^ bioMérieux SA, Marcy-l’Etoile, France, and QIAStat-Dx gastrointestinal panel, QIAGEN, Hilden, Germany, respectively); both assays simultaneously amplify more than 20 different targets for the most common pathogens causing infectious diarrhoea, including viruses, bacteria, and parasites. Subsequently, standard methods (direct microscopy, Gram staining and culture on selective media for 24 h at 37 °C) were performed: MacConkey II Agar, MacConkey Agar with sorbitol, Hektoen Enteric Agar (Becton Dickinson, Inc., Sparks, MD, USA), and Thiosulfate Citrate Bile Sucrose Agar (T.C.B.S. Biolife Italiana, Monza, Italy) were used for culture. The identification of *V. cholerae* was confirmed, with a score ≥ 2, by Maldi-TOF mass spectrometry on plate-cultured colonies (Bruker Daltonik GmbH, Bremen, Germany). The antibiotic susceptibility test was performed using the MIC gradient strip test (Liofilchem, Roseto degli Abruzzi, Italy) and interpreted according to the European Committee on Antimicrobial Susceptibility Testing guidelines (EUCAST; http://www.eucast.org, accessed on 8 April 2022).

### 2.2. Serological and Molecular Characterization

Both isolates were sent to the Regional Reference Laboratory for foodborne pathogens of human origin (LRPTAU) of the Experimental Zooprophylactic Institute of Latium and Tuscany (IZSLT), central division of Rome, according to regional regulations. The isolates were tested using end point PCR for the detection of potentially enteropathogenic *V. cholerae* according to the ISO 21872-1: 2017. The isolates were then sent to Superior Institute of Health (ISS) to perform serotype identification. The strains were streaked on T.C.B.S. cholera medium (Oxoid, UK), on CHROMagar Vibrio (CHROMagar^TM^, Paris, France) and Tryptone Soya Agar (T.S.A. Oxoid, UK). O serotyping analysis was performed using serological slide agglutination with commercial anti-*V. cholerae* O1, anti-*V. cholerae* O139, anti-O1 Inaba and anti-O1 Ogawa antisera (Denka Seiken Co., LTD, Tokyo, Japan). Furthermore, PCR for the presence of the classical cholera toxin and Haitian types of CtxB gene was carried out [17].

### 2.3. Whole Genome Sequencing and Bioinformatics Analysis

Genomic DNA was extracted with the automatic extraction system QIAsymphony (QIAGEN, Hilden, Germany) for whole genome sequencing analysis. Libraries were prepared using Nextera XT DNA Library Prep and pair-end (2 × 250 bp) run with a MiSeq sequencer (Illumina, CA, USA).

Raw reads quality was assessed using Fast QC (v0.11.5) [18] and low-quality reads and adapters were trimmed using Trimmomatic (v0.39) [19] with the following quality filters: minimum quality of Q30, a window size of 10 with Q20 as average quality, and a minimum length read of 50 bp. The high quality reads were de novo assembled into contigs using SPAdes (v3.13.0) with the careful option on [20], draft assemblies were improved using Pilon (v1.23) [21] and contigs shorter than 500 bp were removed [22]. The assembly quality was assessed with QUAST (v5.0.2) [23]. In silico subtyping was performed with Multi-locus Sequence Typing (MLST v2.11) [24] that used the seven housekeeping genes (*adk, gyrB, mdh, metE, pntA, purM, and pyrC*). Antimicrobial resistance genes were identified using BLAST (Camacho et al., 2009) based on the ResFinder database [25] (last access 3/10/2022) and using Comprehensive Antibiotic Resistance Database (CARD, [26]) with the tool Resistance Gene Identifier (RGI). Both methods were used with a threshold of ≥95% identity. VicPred (*Vibrio cholerae* Genotype Prediction Tool) [27] (accessible on http://vicpred.hanyang.ac.kr/, accessed on 1 September 2022) was used for the prediction of cholera toxins (CTX), Vibrio pathogenicity island (VPI) and Vibrio seventh pandemic island (VSP) genetic elements. The characterization of the gene responsible for the determination of the serotype (*wbeT*) was performed to evaluate the presence of mutations, insertions or deletions that could cause the gene disruption and the inactivation of its product(an S-adenosylmethionine-dependent methyl transferase) [28]. The insertion described in the results was identified using the Blast tool [29] implemented in the IS finder [30].

Similar Genome Finder Service [31] was used to find similar public genomes on BV-BRC platform [32], using the GenBank public genome database [33] and the reference genome database. The NCBI Reference Sequence Database is a comprehensive, non-redundant and well-annotated set of reference genome sequences [34]. On the same platform, the Phylogenetic Tree Building Service was used to construct Maximum Likelihood phylogenetic trees with the most similar genomes of both databases previously described [35,36]. For the phylogenetic analysis with the most similar genome found in all public databases, the closer one hundred were selected and three internal reference strains were added. Five strains isolated in previous years in different countries were used as outgroups (CP028827–CP028828, Bangladesh, 1975; PQBQ00000000, Russia, 1972; CP025936–CP025937, USA, 1992; CWRQ00000000, Vietnam, 2003; and JIDH00000000, India, 1941). The Phylogenetic Tree Building Service was performed using the RaxML tool [37] with 500 or 1000 single copy genes partitioned, and a rapid bootstrap of 100 [38]. The trees were visualised using the tool FigTree [39].

### 2.4. Epidemiological Investigation and Food Sample Collection

Following the diagnosis of a cholera case, the Local Health Authorities (LHA) immediately started an epidemiological investigation, taking samples of the food brought back home by the patient. They promptly contacted the patient’s relatives while he was in a serious condition in hospital, and conducted a thorough epidemiological interview. An interview with the patient was conducted as soon as his clinical condition allowed, which led to acquiring further details on general habits, meals and water consumed in the period before the onset of symptoms. The food samples collected by LHA were sent to the Food Microbiology Unit laboratories of IZSLT. Analyses for detection of *V. cholerae* were performed according to ISO 21872-1: 2017.

## 3. Results

### 3.1. Case Presentation and Epidemiological Investigation

We report the case of a 41-year-old patient, with history of a recent one-month stay in Cumilla, a rural area 100 km southeast from Dhaka, Bangladesh. The flight had a stopover in Saudi Arabia and landed in Rome Fiumicino airport on 3 April 2022. The patient reported nausea on the same day, followed by diarrhoea with watery stools and vomiting; he never presented fever. On 5 April he went to the Emergency Department of the “Policlinico Casilino” Hospital where his blood and stool tests were carried out. The blood test showed the following results: leukocytes 17.220/μL, haemoglobin 21 g/dL (hematocrit 61.9%), procalcitonin 0.83 ng/mL, and RCP 21.2 mg/L. The patient had acute renal failure (creatinine 2.08 mg/dL and eGFR38 mL/min). A Filmarray gastrointestinal panel resulted positive for *V. cholerae* and norovirus. The strain of *V. cholerae* was isolated on a specific culture media from the patient’s stools. The SARS-CoV-2 nasopharyngeal swab for the *N* gene and the *Orf1ab* region (Ustar Biotechnologies Hangzhou, China) was negative and the chest CT scan revealed no signs of pneumonia. The patient was referred to the National Institute for Infectious Diseases (INMI) “Lazzaro Spallanzani” for treatment and further investigations.

The patient had received a full-SARS-CoV-2 vaccine cycle (three doses) and had a negative remote pathologycal history. At admission, he was in severe clinical condition, with more than 20 watery diarrhoeal discharges per day and severe dehydration (low blood pressure, dry skin, hypohydrated mucosae, breaded tongue, and hypo-sphygmic pulses). His blood pressure was 80/50 mmHg, and heart rate was 120 bpm. The patient presented tachypnoea (the respiratory rate was 32 per minute) and anuria. The abdomen was globular due to adiposity and tense due to meteorism but treatable on palpation. The patient was in pain but alert and conscious; no meningeal signs were found. Creatinine was 3.81 mg/dL at admission, 5.98 mg/dL the day after, and CPK was 2148 U/l. A severe metabolic acidosis was found (pH 7.2) with a low level of bicarbonate (HCO_3_ 13.6 Mmol/L). Viral hepatitis markers and HIV test were negative. The SARS-CoV-2 nasopharyngeal molecular swab at admission was positive at high cycle threshold (Ct value 37) with negative anti nucleocapsid IgG and positive anti Spike IgG (504 BAU/mL). Widal tests and blood cultures, performed at admission, resulted negative. Toxin detection for *Clostridium difficile* using the immunochromatographic test was negative.

At admission, a new multiplex PCR performed on stool sample confirmed the positive result for *V. cholerae* and norovirus. The stool culture was positive for *V. cholerae* on 5 April, and negative on 9 April. The phenotypic susceptibility profile of the identified *V. cholerae* strain highlighted antibiotic resistance to ciprofloxacin (MIC 0.38 μg/mL), levofloxacin (MIC 0.5 μg/mL), and trimetoprim/sulfametoxazol (MIC > 32 μg/mL) and showed susceptibility to azithromycin (MIC 1.5 μg/mL), cefotaxime (MIC < 0.016 μg/mL), ceftazidime (MIC 0.38 μg/mL), meropenem (MIC 0.38 μg/mL), piperacillin/tazobactam (MIC 0.5 μg/mL), and tetracycline (NA).

During his hospital stay, the patient received intravenous (Ringer lactate solution of 6000 mL the first day, then gradually reduced) and oral fluid therapy associated with electrolyte correction and sodium bicarbonate to restore normal hydration and acid–base balance. Antibiotic therapy was given intravenously (azithromycin 500 mg daily for three days and ceftriaxone 2 g daily for six days) since the patient had uncontrollable vomiting. On 6 April, the patient’s renal function and clinical conditions improved; the frequency of diarrhoeal discharges gradually decreased and the diarrhoeal syndrome resolved completely on 8 April, when intravenous fluids were discontinued.

During the epidemiological investigation, the patient stated that he ate rice, chicken and potatoes and did not drink tap water but only packaged water in the days before the onset of symptoms. He did not perform ritual baths and always stayed in Cumilla. No family member who had travelled and stayed with the patient (his sister and his nephew) neither his relatives living in Bangladesh presented similar symptoms.

On 7 April, the food sampling in patient’s home was performed. The food collected by the local health authorities included dragon fruit, sliced orange fruit (melon or mango), crushed fruit (probably maracuja), a typical rice flour dessert (Nokshi Pitha) and two different kinds of bakery products. No sample was found positive for *V. cholerae.*

**Figure 1:**
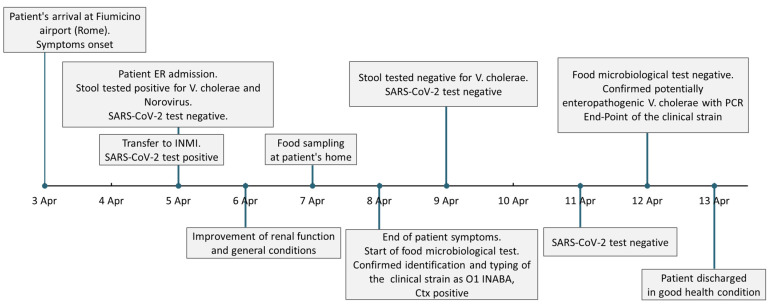
Timeline of the patient’s medical history. Timeline and boxes representing the succession of the main events of the reported cholera case referring to dates. ER—Emergency Department (Policlinico Casilino Hospital, Rome), INMI— National Institute for Infectious Diseases (“Lazzaro Spallanzani”, Rome).

Nasopharyngeal swabs for SARS-CoV-2 RNA performed on 9 and 11 April were negative. On 13 April the patient was discharged in good health with the diagnosis of *V. cholerae* and norovirus enterocolitis complicated by hypovolemic shock, metabolic acidosis, dehydration, acute renal failure and SARS-CoV-2 infection. The timeline of the main events of the case is reported in Figure 1.

### 3.2. V. cholerae Strain Molecular Characterization

Both isolated *V. cholerae* strains were identified as belonging to the O1 Inaba serotype. The end-point PCR for the detection of potentially enteropathogenic *V. cholerae* and PCR for the presence of the cholera toxin gene *CtxB* provided positive results (Haitian variant *Ctx*). The sequence type of the strain was identified as ST69.

The isolate carried the genes (with identity > 95%) for resistance to the following drug classes: carbapenems (*varG*), peptide antibiotics (*almE, almF, and almG*), diaminopyrimidine antibiotics (trimethoprim) (*dfrA1*), sulfonamide antibiotics (sulfamethoxazole) (*sul2*), aminoglycosides (streptomycin) (genes *APH(3″)-Ib* and *APH(6)-Id*), phenicol antibiotics (florfenicol, and chloramphenicol) (genes *floR* and *catB9*), macrolides, fluoroquinolones and penam (CRP).

The genomic region encoding for cholera toxin subunit B, *ctxB7* type (named Haitian variant), showed 100% identity with different genomic sequences of *V. cholerae* O1 Inaba and Ogawa, isolated between 2010 and 2022 in Haiti (GenBank Accession number CP003069-CP003070) [40] and Australia from cases linked to travel to Pakistan (CP102927-CP102928) [41]. Whole genome sequence (WGS) analysis revealed four copies of the repeat (TTTTGAT) in the *ToxR* binding region, as observed in *V. cholerae* Inaba strain CNRVC190243 (OW443147, OW443148, and OW443149) isolated in Yemen in 2019, instead of the five copies of the original Haitian strains of the 2010 outbreak, as described by Kim EJ et al. (2014) [42] (KJ540264). The strain also presented the cholera toxin genetic elements belonging to CTX-1 group (*ace*, *cep*, *ctxA*, *orfU* and *zot* genes). Accessory cholera enterotoxin (Ace) and zonula occludens toxin (Zot) contribute to pathogenesis of *V. cholerae* by inducing changes in the intestinal barrier [43]. The detailed list of the elements of VPI-I, VPI-II, VSP-I and VSP-II is reported in Table S1.

Similar Genome Finder tool detected a high similarity with the reference strain *V. cholerae* O1 biovar ElTor str. N16961 (AE003852, and AE003853), with a Mash distance of 0.93 × 10^−3^, isolated in 1971 in Bangladesh [44].

The phylogenetic analysis of the strain sequence (named *Vibrio cholerae* VUM) with the 100 most similar sequences of the reference database confirmed that the strain belonged to the *V. cholerae* clade (Figure S1).

**Figure 2:**
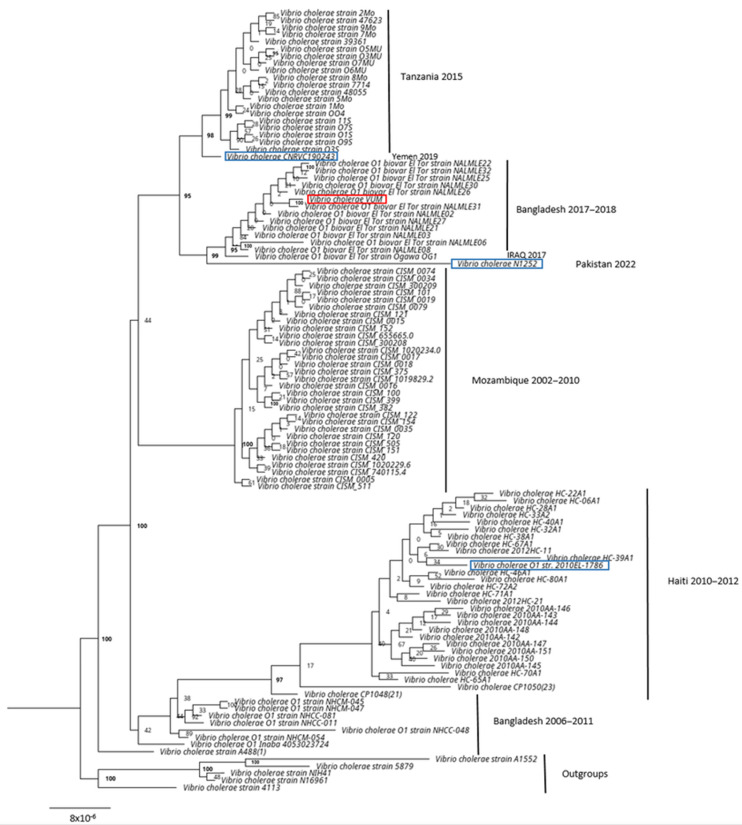
*V. cholerae* maximum likelihood phylogenetic tree based on public database. Maximum Likelihood phylogenetic tree based on the 100 most similar genomes found in the public database with *V. cholerae* clinical strain (highlighted in red, named *Vibrio cholerae* VUM). In blue, the available genomes of reference strains used for the characterization of cholera toxins. In bold, the bootstrap values > 95.

The phylogenetic analysis with the 100 most similar genomes in public databases (Figure 2) showed that the strain was placed in the clade composed by strains of *V. cholerae* O1 biovar ElTor from Bangladesh isolated in 2017–2018 during a seasonal outbreak in the city of Dhaka.

The gene *wbeT*, responsible for the determination of serotype Inaba or Ogawa, was identified in the genome with an insertion of 1261 bp, corresponding to the IS*3*-like element ISV*ch4* family transposase (Figure S1). This evidence confirms the serological identification of the strain as Inaba serotype.

## 4. Discussion

Here, we describe the case of a VFR diagnosed with cholera (imported from Bangladesh), its prompt diagnosis, detailed molecular characterization and clinical management. The concomitant detection of norovirus and SARS-CoV-2 probably did not substantially contribute to the clinical picture because the symptoms were mainly related to cholera. In fact, although norovirus is one of the viral pathogens most frequently recognised using PCR in travellers’ diarrhoea [45], it is possible to find co-infection results, even if at a low frequency, when using highly sensitive multiplex molecular assays, such as those used in our study [46], but it is very difficult to determine the relevance of each of these pathogens detected. To our knowledge, no other cases of coinfection of *V. cholerae* with norovirus have been found in the literature. In this case, the clinical assessment was that the patient’s symptoms were attributable almost exclusively to *V. cholerae*. Cholera is a worldwide infection, and as a communicable disease, can spread across continents within days via air. Even if most people infected with *V. cholerae* do not develop any symptoms, infected subjects can shed bacteria in their faeces for one to ten days after infection, potentially contaminating the environment, and posing a risk of infection to other people [47].

Diagnosis and reporting of cholera cases in non-endemic countries is very rare. Ricaboni et al. described only 14 single imported cases diagnosed in Europe and the United States of America and in the literature published in the years from 1994 to 2018, only three of these cases were observed in Italy, in 2005, 2013 and 2017, respectively [48]. It is noteworthy that the characteristics of the case reported herein are similar to previous cases reported in Italy and other high-income countries. In fact, most of the patients were VFR or travellers and all recovered. In the last five years only another imported case, out of nine sent to ISS for validation, was confirmed as a case of cholera: a strain of *V. cholerae* O1 Inaba serotype, positive for toxin gene variant *CtxB*, was isolated in 2019 from a patient who had eaten food imported from Bangladesh [15,16].

Following the WHO recommendations and definition, when confronted with a patient aged two years or more who developed severe dehydration from acute watery diarrhoea in a non-endemic area, a case of cholera was suspected [3]. The patient was then identified as a probable case in presence of a strong epidemiological link (patient returning from an endemic area). The diagnosis was later microbiologically confirmed, following ECDC case definition (Commission Implementing Decision EU 2018/945, Annex II, 3.7).

Although rapid diagnostic tests can facilitate diagnosis, especially in regions with limited laboratory capacities [49], these assays’ performance ranges widely, showing sensitivities of 58–100% and specificities of 60–100%. In our case, both the multiplex PCR assays showed high values of sensitivity and specificity for *V. cholerae*. In particular, for the FilmArray^®^ gastrointestinal panel, the analytical sensitivity (or limit of detection LoD) reported by the manufacturer is 100% for a LoD of 8 × 103 cells/mL and the specificity is 99.9% (95% Confidence Interval 99.6–100%). For the QIAstat-Dx^®^ Gastrointestinal Panel the sensitivity value declared by the manufacturer is 100% (95% Confidence Interval 34.2–100%) with a specificity of 100% (95% Confidence Interval 98.9–100%). Both assays gave concordant results, with a low turnaround time (TAT) (shorter than two hours), which is crucial in confirming an initial suspicion. Bacterial identification by matrix assisted laser desorption/ionization time of the flight mass spectrometry (MALDI-TOF technique), performed on the strains isolated after 24 h of culture confirmed the species identification. [50]. However, rapid diagnostic tests are only able to identify *V. cholerae* species, but not the serogroup, which is crucial for cholera definition. In fact, at present, cultural and serological techniques remain the gold standard for diagnosis and appropriate case notification and management [51], as they allow to identify serogroups O1 and O139, which are responsible for cholera cases. Most importantly the development of next generation sequencing strategies in support of epidemiological investigations for foodborne diseases, allows in-depth characterization of the strain, detection of virulence determinants and the implementation of phylogenetic and outbreak investigations.

Cholera is a treatable disease through prompt administration of an oral rehydration solution and antibiotics in severe cases. Nevertheless, antimicrobial resistance is an emerging problem. Since the 1990′s, antimicrobial resistance to streptomycin, chloramphenicol and cotrimoxazole has been reported [52] and has been associated with genetic modifications. Antimicrobial susceptibility testing of *V. cholerae* isolated strains is recommended because the resistance patterns vary in different countries and across different times [3]. Currently, most strains are sensitive to tetracyclines, with doxycycline being the gold standard treatment. An emerging resistance to azithromycin has been reported in China, India, and Bangladesh [2], but a recent study showed susceptibility of nearly 100% of isolates for azithromycin and ciprofloxacin in Bangladesh throughout the period 2000–2018, except for *V. cholerae* O1 Ogawa in 2010 when the susceptibility to azithromycin was found to be 69% [53]. Multidrug resistance is an emerging problem for *V. cholerae* in Bangladesh too [54]. In our case, although molecular analysis reported the presence of resistance genes for eight different drug classes, phenotypical resistance was shown for only two drug classes, fluoroquinolones and sulfonamides, as partly expected, and this matched the favourable clinical response of the patient to the administered azithromycin. The divergent phenotypic and genotypic antimicrobial profiles was already described in *V. cholerae* Non-O1/Non-O139 [55]. In light of the changing patterns, phenotypic characterization of antimicrobial resistance of the isolates is important to guide the therapeutic strategies.

Molecular characterization allowed the identification of *CtxB7* (or Haitian Cholera toxin) of *V. cholerae* O1 isolate, known as a hypervirulent strain, which together with the presence of all the main virulence factors can be a cause of severe disease and resistance to ciprofloxacin [56], as in our case.

The interruption of the *wbeT* gene (previously called *rfbT*) causes disruption of the gene and the inactivation of the methyltransferase enzyme which catalyses the methylation of the lipopolysaccharides (LPS) [57]. Strains with methylated LPS are identified as Ogawa serotype, whereas strains with a non-methylated LPS (due to inactivation of the enzyme by mutations in the *wbeT* gene) are identified as Inaba serotype strains. In our case, the gene was interrupted by the presence of a transposable element, the ISV*ch4* of IS3 family, different from the one previously described in the Inaba serotypes described by Baddam et al. in 2020 [58]; such an interruption was not described before. The phylogenetic analysis showed that the isolated clinical strain belonged to the clade composed of isolates from the seasonal outbreak that occurred in Dhaka, Bangladesh during 2017–2018 [58], showing a significant high similarity. All isolates belonging to this outbreak cluster were identified as serotype Ogawa. The clinical isolate described in this study could probably descend from this outbreak and may have, over the years, shifted from Ogawa to Inaba. The serotype shift (from Ogawa to Inaba) is considered a frequent phenomenon, already described, which helps the pathogen to escape acquired immunity [59]. The city visited by the patient is about one hundred kilometers away from the capital of Bangladesh, Dhaka. This country, together with Syria, Afghanistan, and Pakistan, had the highest notification rate worldwide in 2022, that is more than 100 per 100,000 persons, as shown in the geographical distribution map provided by ECDC [14]. The high rate of spread, the lapsed time and geographical dispersion are consistent with the *V. cholerae* genomic reconstruction, which may have contributed to the serotype change responsible for the increase in reinfections.

The prompt evaluation of sampled food brought back from the trip allowed to assess further risks for other people and, from an epidemiological point of view, it is of great importance in preventing and limiting the spread of a potentially severe disease in a non-endemic country; it should always be performed as an essential part of the public health policy for the control and management strategy. In addition, the present work emphasises the importance of a concerted management of a serious communicable disease by the network of health institutions (hospitals, microbiology laboratories, regional and national reference laboratories, regional and national competent authorities, and regional and national surveillance centres) to guarantee rapid diagnosis and disease control.

## 5. Conclusions

The above-reported case highlights the need for prompt recognition and diagnosis, early treatment, and proper and safe management of cholera. It is crucial that the disease is rapidly diagnosed and treated even in settings where *V. cholerae* infection rarely occurs. The molecular characterization and whole genome sequencing and the resulting phylogenetic analysis corroborated the epidemiological data as they allowed to classify the strain with respect to those circulating in the patient’s native area. From a diagnostic point of view, we strongly recommend the adoption of rapid cholera screening tests in Emergency Departments’ laboratories and a close cooperation with advanced diagnostic laboratories for a better characterization of *V. cholerae* isolates to support epidemiological investigations and national and international surveillance activities.

## Data Availability

Clinical data are recorded in the electronic medical records of the hospitals that managed the patient. Raw reads can be found in Sequence Read Archive (SRA) at GenBank database (NCBI, www.ncbi.nlm.nih.gov/, accessed on 3 October 2022) under the bioproject PRJNA941149, biosamples SAMN33591056. All microbiological data are available upon request.

## Supplementary Materials

The following supporting information can be downloaded at: https://www.mdpi.com/article/10.3390/tropicalmed8050266/s1, Table S1. List of genomic virulence factors found. The detailed list of the elements of VPI-I, VPI-II, VSP-I and VSP-II found; Figure S1. Visualisation of the gene wbeT. The gene wbeT identified in the genome with an insertion of 1261 bp, corresponding to the IS3-like element ISVch4 family transposase, wbeT primers (WF1-WR1) were used. DR—direct repeat; IRL—left inverted repeat; IRR—right inverted repeat.

## References

[1] Weil A.A., Ryan E.T. (2018). Cholera. Curr. Opin. Infect. Dis..

[2] Kanungo S., Azman A.S., Ramamurthy T., Deen J., Dutta S. (2022). Cholera. Lancet.

[3] Global Task Force on Cholera Control (GTFCC) (2017). Interim Guidance Document on Cholera Surveillance Global Task Force on Cholera Control (GTFCC) Surveillance Working Group.

[4] Faruque S.M., Albert M.J., Mekalanos J.J. (1998). Epidemiology, Genetics, and Ecology of Toxigenic *Vibrio cholerae*. Microbiol. Mol. Biol. Rev..

[5] Chin C.-S., Sorenson J., Harris J.B., Robins W.P., Charles R.C., Jean-Charles R.R., Bullard J., Webster D.R., Kasarskis A., Peluso P. (2011). The Origin of the Haitian Cholera Outbreak Strain. N. Engl. J. Med..

[6] Ramamurthy T., Garg S., Sharma R., Bhattacharya S.K., Balakrish Nair G., Shimada T., Takeda T., Karasawa T., Kurazano H., Pal A. (1993). Emergence of novel strain of Vibrio cholerae with epidemic potential in southern and eastern India. Lancet.

[7] Faruque S.M., Chowdhury N., Kamruzzaman M., Ahmad Q.S., Faruque A.S.G., Salam M.A., Ramamurthy T., Nair G.B., Weintraub A., Sack D.A. (2003). Reemergence of epidemic *Vibrio cholerae* O139, Bangladesh. Emerg. Infect. Dis..

[8] Global Task Force on Cholera Control Ending Cholera a Global Roadmap to 2030. https://www.gtfcc.org/about-cholera/roadmap-2030/.

[9] Ali M., Nelson A.R., Lopez A.L., Sack D.A. (2015). Updated Global Burden of Cholera in Endemic Countries. PLoS Negl. Trop. Dis..

[10] Paul R.C., Faruque A.S.G., ALAM M., Iqbal A., Zaman K., Islam N., Sobhan A., DAS S.K., Malek M.A., Qadri F. (2016). Incidence of severe diarrhoea due to *Vibrio cholerae* in the catchment area of six surveillance hospitals in Bangladesh. Epidemiol. Infect..

[11] Azman A.S., A Lauer S., Bhuiyan T.R., Luquero F.J., Leung D.T., Hegde S.T., Harris J.B., Paul K.K., Khaton F., Ferdous J. (2020). *Vibrio cholerae* O1 transmission in Bangladesh: Insights from a nationally representative serosurvey. Lancet Microbe.

[12] World Health Organization (2003). List of Member States by WHO Region and Mortality Stratum.

[13] World Health Organization Cholera—Cameroon, 16 May 2022. https://www.who.int/emergencies/disease-outbreak-news/item/2022-DON374.

[14] European Centre for Disease Prevention and Control (ECDC) (2022). Weekly Communicable Disease Threats Report, Week 51, 18–24 December 2022.

[15] European Centre for Disease Prevention and Control (ECDC) (2019). Cholera—Annual Epidemiological Report for 2019.

[16] European Centre for Disease Prevention and Control (ECDC) The European Surveillance System (TESSy): Surveillance Atlas of Infectious Diseases. https://atlas.ecdc.europa.eu/public/index.aspx.

[17] Naha A., Pazhani G.P., Ganguly M., Ghosh S., Ramamurthy T., Nandy R.K., Nair G.B., Takeda Y., Mukhopadhyay A.K. (2012). Development and Evaluation of a PCR Assay for Tracking the Emergence and Dissemination of Haitian Variant ctxB in *Vibrio cholerae* O1 Strains Isolated from Kolkata, India. J. Clin. Microbiol..

[18] Andrews S. Babraham Bioinformatics—FastQC a Quality Control Tool for High throughput Sequence Data. http://www.bioinformatics.babraham.ac.uk/projects/fastqc/.

[19] Bolger A.M., Lohse M., Usadel B. (2014). Trimmomatic: A flexible trimmer for Illumina sequence data. Bioinformatics.

[20] Bankevich A., Nurk S., Antipov D., Gurevich A.A., Dvorkin M., Kulikov A.S., Lesin V.M., Nikolenko S.I., Pham S., Prjibelski A.D. (2012). SPAdes: A New Genome Assembly Algorithm and Its Applications to Single-Cell Sequencing. J. Comput. Biol..

[21] Walker B.J., Abeel T., Shea T., Priest M., Abouelliel A., Sakthikumar S., Cuomo C.A., Zeng Q., Wortman J., Young S.K. (2014). Pilon: An Integrated Tool for Comprehensive Microbial Variant Detection and Genome Assembly Improvement. PLoS ONE.

[22] Bushnell B. BBMap. https://sourceforge.net/projects/bbmap/.

[23] Gurevich A., Saveliev V., Vyahhi N., Tesler G. (2013). QUAST: Quality assessment tool for genome assemblies. Bioinformatics.

[24] Seemann T. GitHub—tseemann/mlst: Scan Contig Files against PubMLST Typing Schemes. https://github.com/tseemann/mlst.

[25] Bortolaia V., Kaas R.S., Ruppe E., Roberts M.C., Schwarz S., Cattoir V., Philippon A., Allesoe R.L., Rebelo A.R., Florensa A.F. (2020). ResFinder 4.0 for predictions of phenotypes from genotypes. J. Antimicrob. Chemother..

[26] Alcock B.P., Huynh W., Chalil R., Smith K.W., Raphenya A.R., A Wlodarski M., Edalatmand A., Petkau A., A Syed S., Tsang K.K. (2023). CARD 2023, expanded curation, support for machine learning, and resistome prediction at the Comprehensive Antibiotic Resistance Database. Nucleic Acids Res..

[27] Lee I., Ha S.-M., Baek M., Kim D.W., Yi H., Chun J. (2021). VicPred: A *Vibrio cholerae* Genotype Prediction Tool. Front. Microbiol..

[28] Lebens M., Karlsson S.L., Källgård S., Blomquist M., Ekman A., Nygren E., Holmgren J. (2011). Construction of novel vaccine strains of Vibrio cholerae co-expressing the Inaba and Ogawa serotype antigens. Vaccine.

[29] Zhang Z., Schwartz S., Wagner L., Miller W. (2000). A greedy algorithm for aligning DNA sequences. J. Comput. Biol..

[30] Siguier P., Perochon J., Lestrade L., Mahillon J., Chandler M. (2006). ISfinder: The reference centre for bacterial insertion sequences. Nucleic Acids Res..

[31] Ondov B.D., Treangen T.J., Melsted P., Mallonee A.B., Bergman N.H., Koren S., Phillippy A.M. (2016). Mash: Fast genome and metagenome distance estimation using MinHash. Genome Biol..

[32] Olson R.D., Assaf R., Brettin T., Conrad N., Cucinell C., Davis J.J., Dempsey D.M., Dickerman A., Dietrich E.M., Kenyon R.W. (2023). Introducing the Bacterial and Viral Bioinformatics Resource Center (BV-BRC): A resource combining PATRIC, IRD and ViPR. Nucleic Acids Res..

[33] Sayers E.W., E Bolton E., Brister J.R., Canese K., Chan J., Comeau D.C., Connor R., Funk K., Kelly C., Kim S. (2022). Database resources of the national center for biotechnology information. Nucleic Acids Res..

[34] O’Leary N.A., Wright M.W., Brister J.R., Ciufo S., Haddad D., McVeigh R., Rajput B., Robbertse B., Smith-White B., Ako-Adjei D. (2016). Reference sequence (RefSeq) database at NCBI: Current status, taxonomic expansion, and functional annotation. Nucleic Acids Res..

[35] Davis J.J., Gerdes S., Olsen G.J., Olson R., Pusch G.D., Shukla M., Vonstein V., Wattam A.R., Yoo H. (2016). PATtyFams: Protein Families for the Microbial Genomes in the PATRIC Database. Front. Microbiol..

[36] Edgar R.C. (2004). MUSCLE: Multiple sequence alignment with high accuracy and high throughput. Nucleic Acids Res..

[37] Stamatakis A. (2014). RAxML version 8, a tool for phylogenetic analysis and post-analysis of large phylogenies. Bioinformatics.

[38] Stamatakis A., Hoover P., Rougemont J. (2008). A rapid bootstrap algorithm for the RAxML Web servers. Syst. Biol..

[39] Rambaut A., Drummond A.J. (2012). FigTree.

[40] Reimer A., Domselaar G., Stroika S., Al A.R.R.E., Kent H., Tarr C., Talkington D., Rowe L., Olsen-Rasmussen M., Frace M. (2011). Comparative genomics of *Vibrio cholerae* from Haiti, Asia, and Africa. Emerg. Infect. Dis..

[41] Sim E.M., Martinez E., Blackwell G.A., Pham D., Millan G., Graham R.M.A., Dhakal R., Wang Q., Suliman B., Jennison A.V. (2023). Genomes of Vibrio cholerae O1 Serotype Ogawa Associated with Current Cholera Activity in Pakistan. Microbiol. Resour. Announc..

[42] Kim E.J., Lee D., Moon S.H., Lee C.H., Kim S.J., Lee J.H., Kim J.O., Song M., Das B., Clemens J.D. (2014). Molecular insights into the evolutionary pathway of *Vibrio cholerae* O1 atypical El Tor variants. PLoS Pathog..

[43] Ramamurthy T., Nandy R.K., Mukhopadhyay A.K., Dutta S., Mutreja A., Okamoto K., Miyoshi S.-I., Nair G.B., Ghosh A. (2020). Virulence Regulation and Innate Host Response in the Pathogenicity of *Vibrio cholerae*. Front. Cell. Infect. Microbiol..

[44] Heidelberg J.F., Eisen J.A., Nelson W.C., Clayton R.A., Gwinn M.L., Dodson R.J., Haft D.H., Hickey E.K., Peterson J.D., Umayam L. (2000). DNA sequence of both chromosomes of the cholera pathogen *Vibrio cholerae*. Nature.

[45] Lääveri T., Antikainen J., Mero S., Pakkanen S.H., Kirveskari J., Roivainen M., Kantele A. (2021). Bacterial, viral and parasitic pathogens analysed by qPCR: Findings from a prospective study of travellers’ diarrhoea. Travel Med. Infect. Dis..

[46] Guan H., Zhang J., Xiao Y., Sha D., Ling X., Kan B. (2016). Evaluation of PCR Based Assays for the Improvement of Proportion Estimation of Bacterial and Viral Pathogens in Diarrheal Surveillance. Front. Microbiol..

[47] Mosley J.F., Smith L.L., Brantley P., Locke D., Como M. (2017). Vaxchora: The First FDA-Approved Cholera Vaccination in the United States. Pharm. Ther..

[48] Ricaboni D., Bozzoni M., Riario Sforza G.G., Rimoldi S.G., Antinori S. (2019). A case of severe cholera imported from Bangladesh to Italy, 2017. Travel Med. Infect. Dis..

[49] Muzembo B.A., Kitahara K., Debnath A., Okamoto K., Miyoshi S.-I. (2022). Accuracy of cholera rapid diagnostic tests: A systematic review and meta-analysis. Clin. Microbiol. Infect..

[50] Dieckmann R., Strauch E., Alter T. (2010). Rapid identification and characterization of Vibrio species using whole-cell MALDI-TOF mass spectrometry. J. Appl. Microbiol..

[51] Centers for Disease Control and Prevention Cholera—Vibrio cholerae Infection. https://www.cdc.gov/cholera/diagnosis.html.

[52] Ghosh A., Ramamurthy T. (2011). Antimicrobials & cholera: Are we stranded?. Indian J. Med. Res..

[53] Parvin I., Shahunja K.M., Khan S.H., Alam T., Shahrin L., Ackhter M.M., Sarmin M., Dash S., Rahman M.W., Bin Shahid A.S.M.S. (2020). Changing Susceptibility Pattern of *Vibrio cholerae* O1 Isolates to Commonly Used Antibiotics in the Largest Diarrheal Disease Hospital in Bangladesh during 2000–2018. Am. J. Trop. Med. Hyg..

[54] Garbern S.C., Chu T.-C., Yang P., Gainey M., Nasrin S., Kanekar S., Qu K., Nelson E.J., Leung D.T., Ahmed D. (2021). Clinical and socio-environmental determinants of multidrug-resistant vibrio cholerae 01 in older children and adults in Bangladesh. Int. J. Infect. Dis..

[55] Lepuschitz S., Baron S., Larvor E., Granier S.A., Pretzer C., Mach R., Farnleitner A.H., Ruppitsch W., Pleininger S., Indra A. (2019). Phenotypic and Genotypic Antimicrobial Resistance Traits of *Vibrio cholerae* Non-O1/Non-O139 Isolated from a Large Austrian Lake Frequently Associated with Cases of Human Infection. Front. Microbiol..

[56] Kumar P., Yadav P., Deshmukh D.G., Bulle P.A., Singh D., Singh N., Sharma K.K., Jain M., Ingole K.V., Goel A.K. (2017). *Vibrio cholerae* O1 with ctxB7 variant genotype acquired qnrVC mediated ciprofloxacin resistance in Yavatmal, India. Clin. Microbiol. Infect..

[57] Wang J., Villeneuve S., Zhang J., Lei P.-S., Miller C.E., Lafaye P., Nato F., Szu S.C., Karpas A., Bystricky S. (1998). On the antigenic determinants of the lipopolysaccharides of *Vibrio cholerae* O:1, serotypes Ogawa and Inaba. J. Biol. Chem..

[58] Baddam R., Sarker N., Ahmed D., Mazumder R., Abdullah A., Morshed R., Hussain A., Begum S., Shahrin L., Khan A.I. (2020). Genome Dynamics of *Vibrio cholerae* Isolates Linked to Seasonal Outbreaks of Cholera in Dhaka, Bangladesh. mBio.

[59] Alam M.T., Ray S.S., Chun C.N., Chowdhury Z.G., Rashid M.H., De Rochars V.E.M.B., Ali A. (2016). Major Shift of Toxigenic *V. cholerae* O1 from Ogawa to Inaba Serotype Isolated from Clinical and Environmental Samples in Haiti. PLoS Negl. Trop. Dis..

