# Supplementary material for: A Cholera Case Imported from Bangladesh to Italy: Clinico-Epidemiological Management and Molecular Characterization in a Non-Endemic Country
Source: Trop Med Infect Dis. 2023 May 6;8(5):266. doi: 10.3390/tropicalmed8050266 (PMC10221301; doi:10.3390/tropicalmed8050266)
Supplement: Supplementary file 1 [file tropicalmed-08-00266-s001.zip › Supplementary Figure S1.pdf]

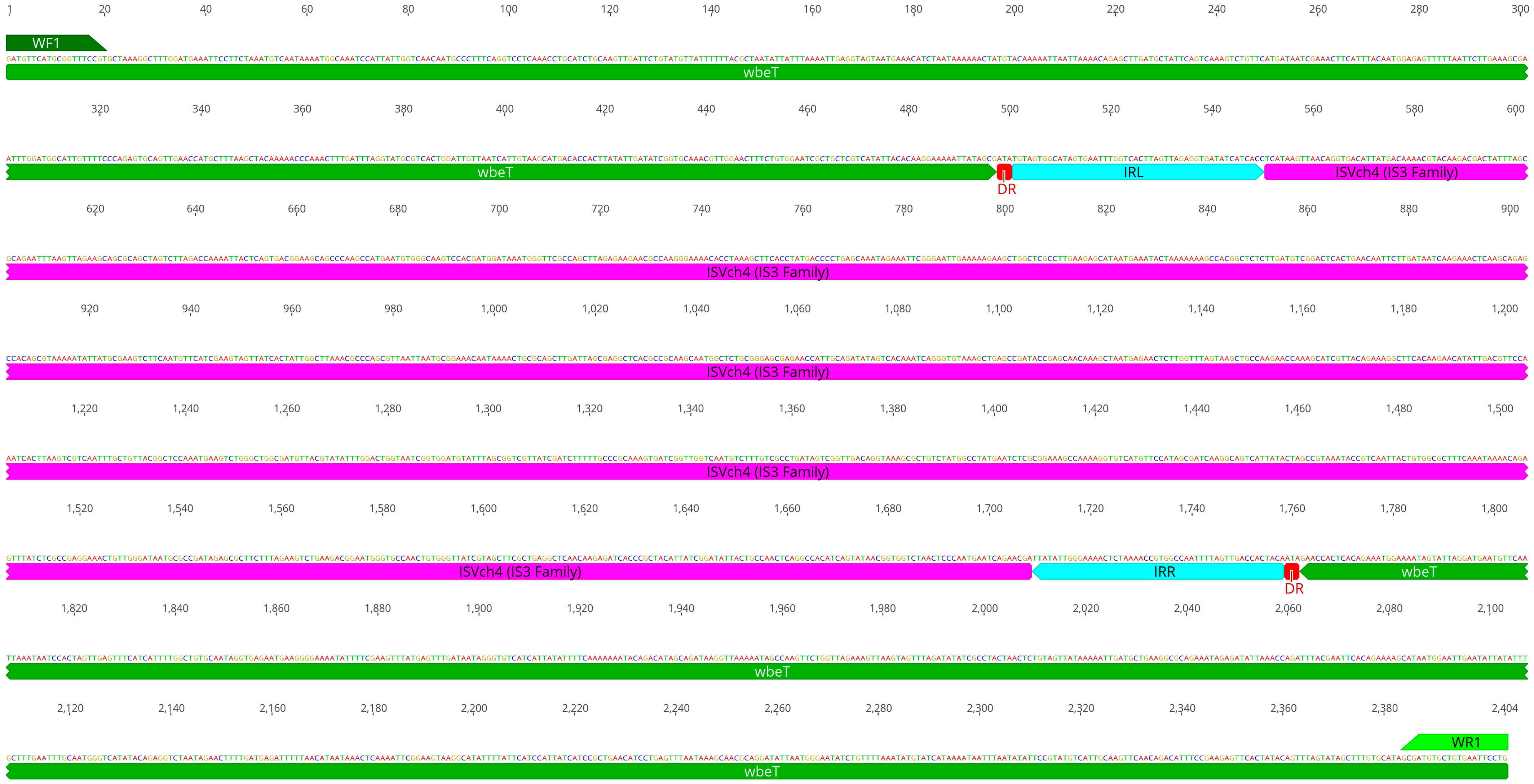

Figure S1. Visualisation of the gene wbeT. The gene wbeT identified in the genome with an insertion of 1261 bp, corresponding to the IS3-like element ISVch4 family transposase, wbeT primers (WF1–WR1) were used. DR–direct repeat; IRL–left inverted repeat; IRR–right inverted repeat.
